# Identification and Antimicrobial Resistance of Bacteria Isolated from Probiotic Products Used in Shrimp Culture

**DOI:** 10.1371/journal.pone.0132338

**Published:** 2015-07-06

**Authors:** Gazi Md. Noor Uddin, Marianne Halberg Larsen, Henrik Christensen, Frank M. Aarestrup, Tran Minh Phu, Anders Dalsgaard

**Affiliations:** 1 Department of Veterinary Disease Biology, Faculty of Health and Medical Sciences, University of Copenhagen, Copenhagen, Denmark; 2 National Food Institute, Technical University of Denmark (DTU), Lyngby, Denmark; University of Aveiro, PORTUGAL

## Abstract

Probiotics are increasingly used in aquaculture to control diseases and improve feed digestion and pond water quality; however, little is known about the antimicrobial resistance properties of such probiotic bacteria and to what extent they may contribute to the development of bacterial resistance in aquaculture ponds. Concerns have been raised that the declared information on probiotic product labels are incorrect and information on bacterial composition are often missing. We therefore evaluated seven probiotics commonly used in Vietnamese shrimp culture for their bacterial species content, phenotypic antimicrobial resistance and associated transferable resistance genes. The bacterial species was established by 16S rRNA sequence analysis of 125 representative bacterial isolates. MIC testing was done for a range of antimicrobials and whole genome sequencing of six multiple antimicrobial resistant *Bacillus* spp. used to identify resistance genes and genetic elements associated with horizontal gene transfer. Thirteen bacterial species declared on the probiotic products could not be identified and 11 non-declared *Bacillus* spp. were identified. Although our culture-based isolation and identification may have missed a few bacterial species present in the tested products this would represent minor bias, but future studies may apply culture independent identification methods like pyro sequencing. Only 6/60 isolates were resistant to more than four antimicrobials and whole genome sequencing showed that they contained macrolide (*erm*D), tetracycline (*tet*L), phenicol (*fex*A) and trimethoprim (*dfr*D, *dfr*G and *dfr*K) resistance genes, but not known structures associated with horizontal gene transfer. Probiotic bacterial strains used in Vietnamese shrimp culture seem to contribute with very limited types and numbers of resistance genes compared to the naturally occurring bacterial species in aquaculture environments. Approval procedures of probiotic products must be strengthened through scientific-based efficacy trials and product labels should allow identification of individual bacterial strains and inform the farmer on specific purpose, dosage and correct application measures.

## Introduction

Aquaculture is the fastest growing animal protein production sector worldwide and Asia contributes annually with about 90% of the global seafood production [1]. Shrimp production in the Mekong Delta of Vietnam alone was 358,477 tons in 2012 accounting for 75% of total shrimp production and 90% of the total area in Vietnam used for shrimp farming [2]. The intensive culture and rapid expansion of shrimp farming in Vietnam and elsewhere have been negatively affected by various diseases, e.g. White Spot Syndrome Virus (WSSV), Yellow Head Virus (YHV), White Feces Syndrome (WFS) and Early Mortality Syndrome (EMS) [3] and water quality problems [4]. In particular tiger shrimp (*Penaeus monodon*) are prone to such diseases [5] and this has been the main driving factor for farmers to change from black tiger to white leg shrimp (*Litopenaeus vannamei*).

A wide range of antimicrobials, disinfectants and other compounds (e.g. nutritional feed supplements) are used to prevent and control shrimp diseases as wells as improving pond water quality. Rico et al. [6] reported that 19% (n = 32) of intensive shrimp farms in Vietnam used oxytetracycline, ciprofloxacin and/or enrofloxacin to treat mainly Early Mortality Syndrome (EMS)/Acute Hepatopancreatic Necrosis Syndrome (AHPNS). The popularity of probiotic usage in shrimp aquaculture has grown worldwide, mainly because farmers often experience limited effect of antimicrobial treatment, but also because of continued problems and reports of antimicrobial residue findings in exported shrimp. Probiotic bacterial strains are anticipated to inhibit pathogens by colonization of the gut-intestinal environment and production of compounds that have a biocidal effect on shrimp pathogens [7]. Furthermore, probiotic bacteria incorporated into shrimp feed may enhance uptake of various nutrients and increase yield [8]. The popularity of probiotic use is highlighted by a recent study where 91% of surveyed shrimp farmers used probiotics [6]. Among these, 84% applied probiotic products directly into the pond water to improve water quality and to reduce environmental stress whereas 16% of farmer’s mixed probiotics with pelleted feed. Rico et al. [6] further reported that *Bacillus subtilis*, *B*. *licheniformis*, *B*. *thuringiensis* and *Lactobacillus acidophilus* were the main bacterial species included in probiotic products used in Vietnamese shrimp culture with bacterial species composition and concentration normally listed on the product labels. However, aquaculture farmers are generally uncertain about the effect of the many different types of marketed probiotics, e.g. those used as feed supplement, whereas a change in water colour after application of probiotics is seen as a sign of improved water quality (Tran Minh Phu, unpublished data).

In contrast to antimicrobials, it is generally believed that probiotics do not play a role in the development of resistance among aquatic animal bacterial pathogens or the general aquatic microflora. Resistance developed through antimicrobial selection pressure may spread by different mechanisms, e.g. horizontal gene transfer via plasmids and other genetic elements [9]. Probiotic bacterial strains used in livestock production have actually been found to contain tetracycline resistance genes [10]. Also, the presence and expression of resistance genes located on plasmids and transposons in *Lactobacillus* spp. and *Bacillus* spp. used as probiotics in foods have been reported [11, 12]. In recent guidelines provided by authorities approving the use of antimicrobials it is clearly stated that the presence of antimicrobial resistance in probiotic bacterial strains are not allowed [13]. Furthermore, bacterial species composition and a measurable beneficial effect and the associated mechanism(s) accounting for such effects seem often not documented for many marketed probiotic products, e.g. the documentation to have probiotic approved for use in aquaculture seem also to vary between countries.

With reference to the increasing use of probiotics in shrimp and other types of aquaculture, the objective of this study was to determine the bacterial species composition in probiotic products commonly used in Vietnamese shrimp culture. Furthermore, the phenotypic antimicrobial susceptibility and genetic basis for antimicrobial resistance in the isolated probiotic strains was determined to assess likelihood of possible transfer of antimicrobial resistance.

## Materials and Methods

### Probiotic Products

Seven probiotic products commonly used in Vietnamese shrimp culture were purchased from aquaculture chemical shops in Soc Trang and Bac Lieu province in the Mekong Delta [14]. Detailed information about the products is shown in Table 1. No specific permission was required by any authority to collect the probiotic products which were purchased from local chemical shops. Three probiotics (I, III and IV) were marketed as feed supplements and four products (II, V, VI and VII) for water treatment in shrimp ponds. Four probiotics were in powder form (I, III, IV, VII) and three contained pellets (II, V, VI). Two probiotic products were imported (III, VI) and distributed by Vietnamese companies while the remaining products were produced and distributed by local companies. Information on product labels was evaluated, e.g. description of contents and formula; information of bacterial genus and species and their concentrations; expiry date, and instruction and health protective measures of use. After purchase, probiotics were stored at room temperature similar to storage conditions in the chemical shops and transported to the University of Copenhagen, Denmark for bacteriological analysis. The products were analysed at least one year before the declared date of expiration.

**Table 1:** Information about the seven probiotic products analyzed.

Product code	Source origin	Declared content	Concentrations (CFU/kg)	Translation of declared information on function of product	Declared dose and application	Package weight	Expiry date	Form	Lot number
I	Vietnam	*Bacillus subtilis*	1.5 × 10^2^	Provide digestive enzymes and useful bacteria to improve feed digestibility in shrimp; improve the efficiency of feed use.	Use 3-5g/1kg feed, apply once a day.	1kg	5/3/2012 to 4/3/2014	Powder	TP810091
		*Pediococcus acidilactici*	1.2 × 10^11^						
		*Lactobacillus acidophilus*	2 × 10^10^						
		*Saccharomyces cerevisiae*	1.2 × 10^11^						
		Phytase	62000 UI/kg						
		Amylase	24000 UI/kg						
II	Vietnam	*B*. *subtilis*	1 × 10^8^	Degrade organic wastes in sediments; improve the water and sediment quality.	Use 1kg for 2000-3000m^3^. Spread into pond in the early morning between 8–9 am.	1kg	14/09/2014	Pellet	TK07-13
		*B*. *licheniformis*	1 × 10^8^						
		*B*. *megaterium*	1 × 10^8^						
III	USA	*B*. *laterrosporus*	0.5 × 10^7^	Provide digestive enzymes to improve feed digestibility in shrimp; prevent and treatment of white feces/stool disease; inhibit growth of *E*.*coli*, *Vibrio*, *Aeromonas*; enhance immune system; increase survival rate; reduce stress.	Use 3g/1kg feed, apply four times a day. Dilute product with 50mL of water then mix it with feed, leave for 10 min and cover by binder (no squid oil use).	454g	3/11/2011 to 5/11/1013	Powder	310-2010/DV
		*B*. *amyloliquefaciens*	0.5 × 10^7^						
		*B*. *megaterium*	1.8 × 10^7^						
		*B*. *thuringiensis*	0.2 × 10^7^						
		*B*. *mesentericus*	0.8 × 10^7^						
IV	Vietnam	*L*. *acidophilus*	1 × 10^6^	Provide digestive enzymes to improve feed digestibility in shrimp.	Use 10g/1kg feed, apply once a day. Dilute product with a little of water then mix it with feed, leave for 15 min and bind with “Glue Fish” product.	1kg	13/09/2014	Powder	TT06-35
		*L*. *sporogenes*	1 × 10^6^						
		*B*. *subtilis*	1 × 10^6^						
		Amylase	1000 UI/kg						
		Protease	500 UI/kg						
V	Vietnam	*B*. *circulans*	0.5 × 10^6^	Degrade organic waste in shrimp pond sediment; absorb toxic gas NH_3_, NO_3_^-^, NO_2_^-^, H_2_S, CH_4_; clean pond bottom; enhance feed intake of shrimp.	Use 900g for 10,000 m^3^. Spread into pond without any dilution with water; apply especially in dirty sediment areas of the pond. Apply once at five days intervals at 8–10 am.	900g	15/4/2011 to 15/4/2013	Pellet	7423479
		*B*. *azotoformans*	0.2 × 10^6^						
		*B*. *pantothenticus*	0.4 × 10^6^						
		*B*. *licheniformis*	1.2 × 10^6^						
		*B*. *thuringiensis*	0.6 × 10^6^						
		*B*. *coagulans*	0.5 × 10^6^						
		*B*. *laterrosporus*	0.2 ˣ 10^6^						
VI	India	*B*. *subtilis*	9 × 10^9^	Degrade organic waste in shrimp pond sediments; improve water and sediment quality.	At stocking densities <20 shrimp/m^2^ apply 40kg/ha before stocking; apply 10kg/ha twice a month after stocking; At stocking densities >20 shrimp/m^2^ apply 50kg/ha before stocking; apply 10kg/ha twice a month after stocking then 15kg/ha and 20kg/ha for the following months. Spread into pond in the early morning between 8–10 am.	5kg	26/8/2011 to 15/8/2016	Pellet	W110037
		*L*. *helveticus*	8 × 10^9^						
		*L*. *lactic*	1 × 10^9^						
		*Nitrosomonas* spp.	6 × 10^7^						
		*Nitrobacter* spp.	6 × 10^7^						
		*Pseudomonas denitrificans*	6 × 10^7^						
		*Alcaligenes denitrificans*	6 × 10^7^						
		*B*. *licheniformis*	1 × 10^6^						
		*S*. *cerevisiae*	5 × 10^5^						
		SiO_2_, MgO, P_2_O_5_, FeSO_4_, CuSO_4_, MnSO_4_, CaO, V_2_O_3_, CoSO_4_	330g, 100g, 100g, 1g, 0.8g, 0.6g, 0.45g, 0.4g, 0.2g in 1kg						
VII	Vietnam	*B*. *subtilis*	1.5 × 10^9^	Degrade organic waste in shrimp pond sediments; improve water and sediment quality; absorb toxic gases like NH_3_, H_2_S; improve water color, e.g. change color from green or dark red to a good water color; increase water oxygen level.	Use 227g/5000m^3^, apply once for 7 days. Incase dirty water pond, apply 227g/3200m^3^; green or dark red water color, apply 227g/1600m^3^; cyanophyta and harmful algae, apply 227g/1600m^3^	227g	4/10/2014	Powder	SJ120401
		*L*. *acidophilus*	1.6 × 10^7^						
		*S*. *cerevisiae*	1.3 × 10^7^						
		*Aspergillus niger*	1.1 × 10^7^						

### Isolation of Bacterial Strains

Based on the product labels of the seven products the following bacterial species were declared: *Bacillus licheniformis*, *B*. *subtilis*, *B*. *megaterium*, *B*. *laterrosporus*, *B*. *amyloliquefaciens*, *B*. *thuringiensis*, *B*. *mesentericus*, *B*. *circulans*, *B*. *azotoformans*, *B*. *pantothenticus*, *B*. *coagulans*, *Lactobacillus acidophilus*, *Pediococcus acidilactici*, *L*. *helveticus*, *L*. *lactic*, *L*. sporogenes, *Nitrobacter* spp., *Nitrosomonas* spp., *Alcaligenes denitrificans*, and *Pseudomonas denitrificans* (Tables 1 and 2). The microbiological analysis done was qualitative to identify the bacterial species as the aim was not to determine concentration of the individual probiotic strains. Tenfold dilutions were prepared for each probiotic products in peptone water (0.1% wt/vol). The products in pellet form were dissolved in peptone water (0.1% wt/vol) using a magnetic stirrer. For the isolation of *Bacillus* spp., tenfold dilutions were spread on blood agar (CM 0055, Oxoid, Basingstoke, Hampshire, England) directly and after a spore activating heat treatment at 85°C for 10 min. Plates were incubated at 30°C for 24 ± 3 h. *Pseudomonas denitrificans* was also recovered on blood agar following incubation at 30°C for 24 ± 3 h. *Lactobacillus* spp. and *Pediococcus acidilactici* were isolated on blood agar, de Man Rogosa and Sharpe (MRS) agar (CM 0361, Oxoid), and All Purpose Tween (APT) agar (1.10453.0500, Merck, Darmstadt, Germany) following incubation at 30°C for 72 h in microaerophilic/anaerobic atmospheres. We did not aim to isolate *Nitrobacter* spp., *Nitrosomonas* spp. and *Alcaligenes denitrificans* as only one product declared to contain these bacteria, but also because of the long incubation period (one to several months for Nitrobacter) required for their isolation.

**Table 2:** Bacterial species isolated from probiotic products after incubation on blood agar for 24 h at 30°C and subsequent identification by 16S rRNA analysis and their susceptibility to antimicrobials.

Probiotic	Bacterial species	Bacterial species identified				Antimicrobial resistance^i^
(purpose of use)	declared on label	ID. no.	Species	Colony morphology (color, surface, margin, size in mm)	Accession no. (%) ^d^	
I	*B*. *subtilis*	1	*B*. *licheniformis* ^g^	Opaque, smooth, fine, 3	FN666245^b^ (100)	CHL, CLI, ERY, PEN, TET, SXT
(feed supplement)	*P*. *acidilactici*	2	*B*. *licheniformis*	Opaque, smooth, fine, 4	FN666245^b^ (100)	-^h^
	*L*. *acidophilus*	3	*B*. *subtilis*	Brown, rough, irregular, 2	AP012496^b^ (100)	CLI
		4	*B*. *amyloliquefaciens*	Dull, rough, irregular, 2	HQ407277 ^b^ (100)	
		5	*B*. *subtilis ss inaquasorum*	Dull, rough, irregular, 2	EU138467^a^ (100)	-^h^
		6	*B*. *subtilis ss subtilis*	Clear, wrinkled, irregular, 2	KC179631^b^ ^c^ (100)	
		55	*B*. *amyloliquefaciens ss plantarum*	White, wrinkled, irregular, 3	CP000560^a^ (100)	
		56	*B*. *sonorensis*	Clear, rough, irregular, 3	AF302118^a^ (100)	-^h^
		57	*B*. *sonorensis*	Clear, rough, irregular, 4	AF302118^a^ (100)	-^h^
		58	*B*. *methylotrophicus*	Dull, rough, irregular, 3	EU194897^a^ (100)	AMP, CHL, ERY
		59	*B*. *subtilis ss subtilis*	Dull, rough, irregular, 3	ABQL01000001^a^ (100)	AMP, CHL
		60	*B*. *licheniformis*	Clear, rough, irregular, 3	AE017333^a^ (100)	CHL, CLI, PEN
		61	*B*. *cereus* ^f^	Glistening, granular flat, irregular, hemolysis, 2	AE017194^b^ (100)	-^h^
		62	*B*. *safensis*	Dull, rough, irregular, 2	AF234854^a^ (100)	CLI
		63	*B*. *sonorensis*	Dull, rough, irregular, 3	AF302118^a^ (100)	CHL, CLI, PEN
		96	*B*. *cereus* ^f^	Glistening, granular-flat, irregular, hemolysis, 6	AP007209^b^ (99)	-^h^
		97	*B*. *cereus* ^f^	Glistening, granular-flat, irregular, hemolysis, 5	AP007209^b^ (99)	-^h^
		98	*B*. *cereus* ^f^	Glistening, granular-flat, irregular, hemolysis, 5	AP007209^b^ (99)	AMP, PEN, TET
		99	*B*. *subtilis ss inaquosorum*	Dull, wrinkled, irregular, 3	EU138467^a^ (100)	
		100	*B*. *subtilis*	Dull, wrinkled, irregular, 4	JQ403532^b^ ^c^ (100)	-^h^
		101	*B*. *subtilis ss subtilis*	Dull, wrinkled, irregular, 3	ABQL01000001^a^ (100)	-^h^
		102	*B*. *subtilis*	Dull, wrinkled, irregular, 2	JQ308575^b^ (100)	
		103	*B*. *subtilis*	Dull, wrinkled, irregular, 2	GU826165^b^ (100)	
		104	*B*. *subtilis*	Dull, wrinkled, irregular, 4	GU826165^b^ (100)	-^h^
		105	*B*. *methylotrophicus*	Dull, rough, irregular, 2	EU194897^a^ (100)	-^h^
		106	*B*. *subtilis ss inaquosorum*	Dull, rough, irregular, 2	EU138467^a^ (100)	-^h^
		107	*B*. *subtilis*	Dull, rough, irregular, 3	GU826165^b^ (100)	-^h^
II	*B*. *subtilis*	50	*B*. *amyloliquefaciens* ss *plantarum*	Dull, rough, irregular, 6	CP000560^a^ (100)	PEN
(water treatment)	*B*. *licheniformis*	51	*B*. *amyloliquefaciens* ss *plantarum*	Dull, rough, irregular, 5	CP000560^a^ (100)	-^h^
	*B*. *megaterium*	52	*B*. *amyloliquefaciens* ss *plantarum*	Dull, rough, irregular, 4	CP000560^a^ (100)	-^h^
		53	*B*. *amyloliquefaciens* ss *plantarum*	Dull, rough, irregular, 3	CP000560^a^ (100)	-^h^
		54	*B*. *amyloliquefaciens* ss *plantarum*	White, wrinkled, irregular, 3	CP000560^a^ (100)	-^h^
III	*B*. *laterrosporus*	7	*B*. *amyloliquefaciens*	Opaque, rough, irregular, 2	KC250199^b^ (100)	
(feed supplement)	*B*. *megaterium*	8	*B*. *amyloliquefaciens* ss *plantarum* ^g^	Opaque, rough, irregular, 2	HE617159^b^ (100)	CHL, CLI, ERY, TET
	*B*. *amyloliquefaciens*	9	*B*. *tequilensis*	Frost glass, rough, irregular, 4	HQ223107^a^ (100)	
	*B*. *thuringiensis*	10	*B*. *subtilis*	Frost glass, rough, irregular, 3	CP002468^b^ (100)	
	*B*. *mesentericus*	11	*B*. *tequilensis* ^g^	Frost glass, rough, irregular, 3	KC172053^b^ ^c^ (100)	CHL, ERY, PEN, TET, SXT
		12	*B*. *subtilis* ss *inaquosorum*	Frost glass, rough, irregular, 3	EU138467^a^ (100)	-^h^
		13	*B*. *safensis*	Dull, flat, irregular, 2	AF234854^a^ (100)	CLI
		14	*B*. *amyloliquefaciens* ss *plantarum*	Dull, flat, irregular, 2	CP000560^a^ (100)	-^h^
		15	*B*. *subtilis* ss *inaquosorum*	Dull, rough, irregular, 5	EU138467^a^ (100)	
		16	*B*. *subtilis*	Dull, rough, irregular, 5	CP002468^b^ (100)	-^h^
		17	*B*. *subtilis* ss *inaquosorum*	Dull, rough, irregular, 4	EU138467^a^ (100)	-^h^
		18	*B*. *subtilis* ss *inaquosorum*	Dull, rough, irregular, 4	EU138467^a^ (100)	-^h^
		19	*B*. *tequilensis*	Dull, rough, irregular, 3	JN641294^b^ ^c^ (100)	
		20	*B*. *amyloliquefaciens* ss *plantarum*	Dull, rough, irregular, 3	CP000560^a^ (100)	ERY
IV	*L*. *acidophilus*	21	*B*. *tequilensis*	Dull, flat, irregular, 2	DQ989210^b^ (100)	PEN
(feed supplement)	*L*. *sporogenes*	22	*B*. *subtilis*	Dull, rough, irregular, 4	AP012495^b^ (99)	
	*B*. *subtilis*	23	*B*. *amyloliquefaciens* ss *plantarum*	Dull, rough, irregular, 3	CP005660^b^ (100)	
		24	*B*. *subtilis* ss *subtilis*	Dull, rough, irregular, 3	JQ396173^b^ (100)	
		25	*B*. *stratosphericus*	Dull, flat, irregular, 1	AJ831841^a^ (100)	CLI
		26	*B*. *cereus* ATCC 10987^e^ ^g^	Glistening, granular flat, irregular, hemolysis, 5	AE017194^b^ (99)	AMP, CHL, PEN, SXT
		27	*B*. *cereus* ATCC 10987^e^	Glistening, granular flat, irregular, hemolysis, 4	AE017194^b^ (99)	-^h^
		64	*Kleb*. *pneumoniae* ss *ozaenae*	Shiny, convex, fine, 2	Y17654^a^ (100)	AMP
		65	*Kleb*. *pneumoniae* ss *ozaenae*	Shiny, convex, fine, 2	Y17654^a^ (100)	-^h^
		66	*B*. *megaterium*	Watery, convex, irregular, 2	D16273^a^ (100)	-^h^
		67	*Aerococcus urinaeequi*	White, Elevated, fine, 2	D87677^a^ (100)	AMP. CHL, CLI, ERY, OXA, PEN,SXT
		68	*B*. *amyloliquefaciens* ss *plantarum*	Dull, rough, irregular, 5	CP000560^a^ (100)	-^h^
		69	*B*. *amyloliquefaciens* ss *plantarum*	Dull, rough, irregular, 5	CP000560^a^ (100)	-^h^
		70	*B*. *megaterium*	Dull, rough-flat, irregular, 3	D16273^a^ (100)	CLI
		71	*B*. *safensis*	Dull, rough-flat, irregular, 3	AF234854^a^ (100)	CLI
		72	*B*. *amyloliquefaciens* ss *plantarum*	Dull, rough-flat, irregular, 3	CP000560^a^ (100)	-^h^
		73	*B*. *subtilis* ss *subtilis*	Opaque, rough, irregular, 3	ABQL01000001^a^ (100)	CLI
		74	*B*. *amyloliquefaciens* ss *plantarum*	Opaque, rough, irregular, 3	CP000560^a^ (100)	-^h^
		75	*B*. *methylotrophicus*	Opaque, rough, irregular, 3	EU194897^a^ (100)	CHL
		76	*B*. *cereus* ATCC14578 T^e^	Glistening, rough, irregular, hemolysis, 3	AE017194^b^ (99)	-^h^
		77	*B*. *cereus* ATCC14578 T^e^	Glistening, rough, irregular, hemolysis, 3	AE017194^b^ (99)	-^h^
		108	*Kleb*. *pneumoniae* ss *pneumoniae*	Creamy, convex, fine, 2	AB004753^a^ (100)	AMP
		109	*Kleb*. *pneumoniae*	Creamy, convex, fine, 2	AB641122^b^ (99)	AMP
		110	*Kleb*. *singaporensis*	Creamy, convex, fine, 2	AF250285^a^ (99)	AMP
		111	*B*. *cereus* ATCC 14579^e^	Glistening, flat, irregular, hemolysis, 2	AE016877^b^ (99)	-^h^
		112	*B*. *cereus* ATCC 14579^e^	Glistening, flat, irregular, hemolysis, 3	AE016877^a^ (100)	-^h^
		113	*Kleb*. *pneumoniae* ss *pneumoniae*	Creamy, convex, fine, 2	AB004753^a^ (100)	-^h^
V	*B*. *circulans*	28	*B*. *megaterium*	Dull, rough, irregular, 2	GU252120^b^ (100)	CLI
(water treatment)	*B*. *azotoformans*	29	*B*. *subtilis* ss *subtilis*	Dull, rough, irregular, 3	ABQL01000001^a^ (100)	
	*B*. *pantothenticus*	30	*B*. *tequilensis*	Dull, rough, irregular, 3	HQ154527^b^ (99)	
	*B*. *licheniformis*	31	*B*. *subtilis* ss *subtilis*	Dull, smooth, fine, 4	ABQL01000001^a^ (100)	-^h^
	*B*. *thuringiensis*	32	*B*. *subtilis*	Dull, smooth, fine, 4	HQ336634^b^ (100)	
	*B*. *coagulans*	33	*B*. *licheniformis*	Clear, convex, fine, 1	AE017333^a^ (100)	-^h^
	*B*. *laterrosporus*	34	*B*. *licheniformis*	Clear, convex, fine, 1	AE017333^a^ (100)	-^h^
		35	*B*. *firmus*	Clear, convex, fine, 1	X60616^a^ (100)	CLI, ERY, PEN
		36	*B*. *amyloliquefaciens*	Clear, rough, irregular, 5	CP003838^b^ ^c^ (99)	CLI
		37	*B*. *licheniformis*	Clear, convex, fine, 3	AE017333^a^ (100)	CHL, CLI, ERY
		38	*B*. *licheniformis*	Clear, convex, fine, 3	AE017333^a^ (100)	-^h^
		39	*B*. *subtilis* ss *inaquosorum*	Dull, convex, fine, 3	EU138467^a^ (100)	
		40	*B*. *firmus*	Dull, convex, fine, 4	X60616^a^ ^c^ (100)	-^h^
		41	*B*. *licheniformis*	Clear, convex, fine, 1	AE017333^a^ (100)	-^h^
		42	*B*. *aerius* ^g^	Clear, convex, fine, 1	AJ831843^a^ (100)	CIP, CHL
VI	*B*. *subtilis*	43	*B*. *subtilis* ss *inaquosorum*	Dull, wrinkled, irregular, 3	EU138467^a^ (100	CHL
(water treatment)	*L*. *helveticus*	44	*B*. *vallismortis*	Dull, wrinkled, irregular, 6	AB021198^a^ (100)	
	*L*. *lactic*	45	*B*. *safensis*	Dull, rough-flat, irregular, 3	AF234854^a^ (100)	CHL, CLI
	*Nitrobacter* spp.	46	*B*. *safensis*	Dull, rough-flat, irregular, 3	AF234854^a^ (100)	-^h^
	*Nitrosomonas* spp.	78	*B*. *amyloliquefaciens*	Dull, smooth, fine, 4	CP002927^b^ (100)	
	*A*. *denitrificans*	79	*B*. *safensis*	Dull, rough-flat, irregular, 2	AF234854^a^ (100)	-^h^
	*P*. *denitrificans*	80	*B*. *pumilus*	Dull, rough, irregular, 2	JX860616^b^ ^c^ (100)	CLI
	*S*. *cerevisiae*	81	*B*. *licheniformis*	Clear, rough, regular, 2	AE017333^a^ (100)	-^h^
	*B*. *licheniformis*	82	*B*. *subtilis* ss *inaquosorum*	Clear, rough, irregular, 4	EU138467^a^ (99)	-^h^
		84	*B*. *licheniformis*	Clear, rough, irregular, 2	AE017333^a^ (100)	CHL, CLI
		114	*B*. *amyloliquefaciens*	Opaque, rough, irregular, 4	CP002927^b^ (100)	-^h^
		115	*B*. *subtilis*	Opaque, rough, irregular, 4	JN054738^b^ (100)	
		116	*B*. *subtilis* ss *inaquosorum*	Opaque, rough, irregular, 4	EU138467^a^ (100)	-^h^
		117	*B*. *pumilus*	Dull, wrinkled, irregular, 3	HQ650161^b^ (100)	-^h^
		118	*B*. *pumilus*	Dull, wrinkled, irregular, 3	FJ705814^b^ (99)	-^h^
		119	*B*. *pumilus*	Dull, wrinkled, irregular, 3	AB354235^b^ (100)	-^h^
		120	*B*. *amyloliquefaciens* ss *plantarum*	Dull, wrinkled, irregular, 4	CP000560^a^ (100)	CLI
VII	*B*. *subtilis*	47	*B*. *subtilis*	Frost glass, rough, irregular, 2	JX960641^b^ ^c^ (99)	-^h^
(water treatment)	*L*. *acidophilus*	48	*B*. *amyloliquefaciens*	Frost glass, rough, irregular, 5	CP002927^b^ (100)	TET
	*S*. *cerevisiae*	49	*B*. *licheniformis*	Opaque, rough, regular, 3	AE017333^a^ (100)	-^h^
	*A*. *niger*	85	*B*. *amyloliquefaciens*	Opaque, rough, irregular, 3	CP000560^a^ (100)	CLI, CHL
		86	*B*. *licheniformis*	Opaque, rough, irregular, 3	AE017333^a^ (100)	-^h^
		87	*B*. *sonorensis*	Clear, rough, irregular, 2	AF302118^a^ (100)	CLI, ERY
		88	*B*. *amyloliquefaciens* ss *amyloliquefaciens*	Dull, rough, fine, 3	FN597644^a^ (100)	
		89	*B*. *licheniformis*	Dull, rough, fine, 4	AE017333^a^ (100)	CHL, CLI, ERY, PEN
		90	*B*. *subtilis* ss *inaquosorum*	Frost glass, rough, irregular, 3	EU138467^a^ (100)	CHL, CLI
		91	*B*. *nealsonii* ^g^	Frost glass, flat, irregular, 2	EU656111^a^ (100)	CHL, CIP, ERY, SXT
		92	*B*. *subtilis* ss *inaquosorum*	Brown, flat, irregular, 3	EU138467^a^ (100)	-^h^
		93	*B*. *cereus* ATCC 10987^e^	Glistening, rough, irregular, hemolysis, 3	AE017194^b^ (100)	-^h^
		94	*B*. *cereus* ATCC 10987^e^	Glistening, rough, irregular, hemolysis, 3	AE017194^b^ (100)	-^h^
		95	*B*. *cereus* ATCC 10987^e^	Glistening, rough, irregular, hemolysis, 3	AE017194^b^ (99)	-^h^
		121	*B*. *subtilis* ss *inaquosorum*	Dull, rough, irregular, 2	EU138467^a^ (100)	-^h^
		122	*B*. *vallisomortis*	Dull, rough, irregular, 2	AB021198^a^ (100)	
		123	*B*. *subtilis* ss *inaquosorum*	Dull, rough, irregular, 4	EU138467^a^ (100)	-^h^
		124	*B*. *cereus* ATCC 10987^e^	Glistening, rough, irregular, hemolysis, 4	AE017194^b^ (99)	PEN
		125	*B*. *cereus* ATCC 14579^e^	Glistening, rough, irregular, hemolysis, 3	AF090330^b^ (99)	-^h^
		126	*B*. *cereus* ATCC 14579^e^	Glistening, rough, irregular, hemolysis, 3	AF090330^b^ (99)	-^h^

^a^ Accession number for the Ez-Taxon database.

^b^ Accession number for the GenBank database.

^c^ Unpublished reference.

^d^ Nucleotide similarity in percent.

^e^ *Bacillus cereus* identified by *gyr*B sequencing analysis.

^f^ *Bacillus cereus* identified by MALDI-TOF method.

^g^ Strains characterized by whole genome sequencing.

-^h^ Not tested.

^i^ CLI, clindamycin; CIP, ciprofloxacin; CHL, chloramphenicol; ERY, erythromycin; PEN, penicillin; TET, tetracycline; SXT, trimethoprim/sulfamethoxazole; AMP, ampicillin; GEN, gentamicin; OXA, oxacillin.

The colonies on blood agar were categorised into different types according to their colony characteristics: color, opacity, surface and border structure, diameter size and haemolytic property. Between three to five colonies of each morphology type were randomly selected and sub-cultured on blood agar to obtain pure cultures. The isolates were characterised by the Gram reaction using 3% (wt/vol) potassium hydroxide (Bie and Berntsen, Herlev, Denmark), motility, cytochrome oxidase test (NN-Dimethyl-p-phenylene-diamine dihydrochloride, Remel Europe Ltd., Dartford, UK), and catalase test following the procedures described by Cowan [15]. Loeffler’s polychrome methylene blue stain (McFadyen’s reaction) was used for spore staining and the presence of spores was also studied using phase contrast microscopy. All strains were cultured in Brain Heart Infusion broth (CM 1135, Oxoid) and stored at -80°C with 30% (vol/vol) glycerol until further characterisation.

### Bacterial Identification by 16S rRNA Sequence Analysis

A total of 94 bacterial isolates cultured on blood agar incubated for 24 ± 3 h at 30°C in ambient atmospheric conditions and 31 isolates cultured on blood agar incubated at 30°C in anaerobic conditions for 72 h, were selected as representatives of the different colony morphology types (three to five isolates selected for each colony morphology type) seen after culture of the probiotic products. Identification of the presumptive *Bacillus*, *Klebsiella* and *Aerococcus* isolates were confirmed by 16S rRNA sequence analysis. Total genomic DNA was extracted using the DNeasy Blood & Tissue kit following the manufacturer’s protocol for Gram-positive and Gram-negative bacteria (Qiagen, Hilden, Germany). The universal primer sets described by Weisberg et al. [16] were employed for the sequencing of the 16S rRNA genes (Table 3). Sequencing of the PCR amplicons was done by Macrogen, Inc (Seoul, Korea). The 16S rRNA sequence data were compared with available sequence data in the GenBank and EZ-taxon databases using the BLAST algorithm [17].

**Table 3:** Oligonucleotide primers used for 16S rRNA analysis and detection of the *gyr*B gene.

Species	Primer	Sequence (5´^-^3´)	Target gene
*Bacillus*, *Lactobacillus*	8-27F	AGA GTT TGA TCC TGG CTC AG	16S rRNA
*Bacillus*, *Lactobacillus*	1390–1408 R	TGA CGG GCG GTG TGT ACA A	16S rRNA
*Bacillus*, *Lactobacillus*	786F	GAT TAG ATA CCC TGG TAG	16S rRNA
*Bacillus*, *Lactobacillus*	786R	CTA CCA GGG ATAT CTA ATC	16S rRNA
*Bacillus*, *Lactobacillus*	344R	ACT GCT GCC TCC CGT	16S rRNA
*Bacillus*, *Lactobacillus*	344F	ACG GGA GGC AGC AGT	16S rRNA
*Bacillus*, *Lactobacillus*	785 805 F	GGA TTA GAT ACC CNG GTA GTC	16S rRNA
*Bacillus*, *Lactobacillus*	785 805R	GAC TAC CNG GGT ATC TAA TCC	16S rRNA
*Bacillus*, *Lactobacillus*	37F	GGC TCA GRW YGA ACG C	16S rRNA
*Bacillus cereus*	BCJH-F	TCATGAAGAGCCTGTGTACG	*gyr*B
*Bacillus cereus*	BCJH-1R	CGACGTGTCAATTCACGCGC	*gyr*B
*Bacillus thuringiensis*	BTJH-1F	GCTTACCAGGGAAATTGGCAG	*gyr*B
*Bacillus thuringiensis*	BTJH-R	ATCAACGTCGGCGTCGG	*gyr*B

### Discrimination of *Bacillus cereus* and *B*. *thuringiensis*

*Bacillus cereus* and *B*. *thuringiensis* are highly polyphyletic [18] and have similar genotypic and phenotypic properties [19]. PCR of the gyrase B (*gyr*B) gene [20] and subsequent sequencing was used to differentiate the two species (Table 2) [21, 22]. PCR amplification was performed for 12 isolates under the following conditions: initial denaturation at 94°C for 3 min for 1 cycle, 35 cycles consisting of denaturation at 94°C for 45 sec, annealing at 63°C for 1 min and elongation at 72°C for 1 min, and a final extension at 72°C for 7 min [22]. *B*. *cereus* ATCC 11778 and *B*. *thuringiensis* CCUG 7429T were used as positive controls. Sequencing of the PCR amplicons was done by Macrogen, Inc, South Korea and data were compared with available *gyr*B gene sequence data in the GenBank database using BLAST algorithm. Similarity of *B*. *cereus* and *B*. *thuringiensis* were determined by pair wise comparison using ‘water’ available as EMBOSS program on the EBI server (http://www.ebi.ac.uk/Tools/psa/emboss_water/nucleotide.html).

Because of incomplete sequence data obtained for four isolates the identity of these isolates were confirmed by phenotypic characteristics established using Matrix-assisted laser desorption/ionization-time of flight (MALDI-TOF, Biomerieux, Germany) [23]. In brief, the isolates were identified by using an AXIMA Assurance (Shimadzu-Biotech) MALDI-TOF mass spectrometer machine (Shimadzu-Biotech, Kyoto, Japan). The isolates were prepared and analysed according to the instructions of the manufacturer. Only profiles that passed the auto-quality control with the Launchpad software (version 2.9) (Shimadzu-Biotech, Kyoto, Japan) were used for spectrum accumulation. *Escherichia coli* ATCC 8739 was used as a calibrator and internal identification control in each series of measurements. Raw spectra were automatically processed with the Launchpad software and resulting peak lists were transferred to and analyzed with the SARAMIS software [23]. Comparison of sample spectra to SuperSpectra in the SARAMIS database (version 4.09) was considered significant when a confidence value for a match was at least 75%. All identification results with confidence values of at least 80% were considered as reliable when no conflicts were indicated, i.e., when all significant matches gave exactly the same species or genus, respectively. In case of no significant matches to SuperSpectra, the sample spectra was compared to all reference spectra in the database and all spectra giving matches exceeding the minimum similarity criteria were shown for further manual evaluation.

### Phylogenetic Tree Analysis of *Bacillus* spp.

The regions of the multiple alignment that included the 5' (5´-AGA GTT TGA TCC TGG CTC AG-3´) and 3' end 1390–1408 (TTGTACACACCGCCCGTCA) sequencing primers were trimmed away and strains with identical sequences were only represented once in the phylogenetic analysis. Multiple alignments and neighbour joining phylogenetic analysis including calculation of bootstrap support were done by ClustalX2 [24] and MEGA5 [25] was used for graphical representation of trees. Sequences of less than 1 kb in length were excluded from the multiple alignment and phylogenetic analysis and they were only analysed by Eztaxon-e comparison to sequences of type strains. Species identification of 16S rRNA sequences was based on comparisons to similarity with type strains of species as provided in Eztaxon-e [26] using a threshold of 99%. This will give an error of 5% in the identification [27].

### Antimicrobial Susceptibility Testing

Antimicrobial susceptibility was determined for 65 strains selected among a total of 125 strains identified. The strains were selected based on the criteria that the specimen selected showing the highest nucleotide similarity percentage as compared with the Ez-taxon database and one representative strain from each bacterial species identified in a particular product should be represented. The strains selected included 60 *Bacillus* spp., one *Aerococcus* spp. and four *Klebsiella* spp. strains. No *Lactobacillus*, *Pseudomonas*, and *Pediococcus* were isolated. Antimicrobial susceptibility testing by broth microdilution was done using Sensititre GPALL1F and GN3F panels (Trek Diagnostics System, East Grinstead, UK) according to guidelines from the Clinical and Laboratory Standards Institute (CLSI) [28]. The GPALL1F (Gram-positive) panel included β-lactams [ampicillin (AMP), penicillin (PEN) and oxacillin (OXA) with 2% NaCl] and non-β-lactam antimicrobials [chloramphenicol (CHL), daptomycin (DAP), gentamycin (GEN), linezolid (LZD), rifampicin (RIF), tetracycline (TET), erythromycin (ERY), trimethoprim/sulfamethoxazole (SXT), quinupristin/dalfopristin (Q-D), vancomycin (VAN), levofloxacin (LVX), tigeccycline (TGC), moxifloxacin (MXF), clindamycin (CLI), streptomycin (STR), ciprofloxacin (CIP), and nitrofurantoin (NIT)]. While the GN3F (Gram-negative) panel included β-lactams [ampicillin (AMP), ampicillin/sulbactam (SAM), aztreonam (ATM); cefazolin (CFZ), cefepime (FEP), meropenem (MEM), ertapenem (ETP), cefuroxime (CXM), cefoxitin (FOX), cefpodoxime (CPD), ceftazidime (CAZ), ceftriaxone (CRO), cephalothin (CEF) and ticarcillin/clavulanic acid (TIM) constant 2] and non- β-lactam antimicrobials [amikacin (AMK), gentamicin (GEN), ciprofloxacin (CIP), pipercillin/tazobactam (TZP) constant 4, tobramycin, tigeccycline (TGC) trimethoprim/sulfamethoxazole (SXT) and tetracycline (TET)]. Following 48 h incubation at 30°C [29], the plates were read using Sensititre Vizion System (Trek Diagnostics System) and MIC values were interpreted according to CLSI breakpoints for *Staphylococcus* spp. [29] and *Escherichia coli* [28]. As there are no guidelines breakpoints for *Bacillus* spp. in CLSI guideline and *Bacillus* spp. are mainly fastidious Gram-positive bacteria like *Staphylococcus* spp. therefore the breakpoints of *Staphylococcus* spp. were used as criteria for interpretation. The breakpoints of *E*. *coli* are recommended and used for other Enterobacteriaceae such *Klebsiella* species [28].

### Whole Genome Sequence Analysis to Identify Antimicrobial Resistance Genes

As *Bacillus* spp. were by far the most commonly declared and isolated bacterial species (Table 2), six representative isolates including *B*. *licheniformis*, *B*. *amyloliquefaciens*, *B*. *tequilensis*, *B*. *cereus*, *B*. *aerius*, and *B*. *nealsonii* were selected for whole genome sequencing to determine antimicrobial resistance genes. The isolates were selected based on their multiple antimicrobial resistance patterns mainly to antimicrobials used therapeutically in humans e.g. ciprofloxacin, tetracycline, sulfamethoxazole/trimethoprim, erythromycin and clindamycin. Paired-end Illumina sequencing of the strains was carried out on an Illumina Miseq, following standard Illumina protocols (Illumina, Inc., USA). Available genes in the ResFinder database [30] were BLASTed against the assembled genome, and the best-matching genes were counted as output [30]. The contigs with identified resistance genes were blasted against the NCBI database and the regions surrounding the resistance genes examined for potential genes associated with horizontal transfer.

## Results

### Product Label Information

Information declared on the product label included purpose of use (feed supplement and water treatment) and bacterial species composition as shown in Table 1. The declared concentrations of the individual bacterial strains varied between 10^6^ to 10^12^ cfu/kg, i.e. product I contained 1.5 x 10^12^ *B*. *subtilis*/kg, product II contained 1 x 10^8^ *B*. *subtilis*/*B*. *licheniformis*/*B*. *megaterium*/kg; and product V contained between 0.2–1.2 x 10^6^ of the seven declared *Bacillus* spp. (Table 1). However, based on the serial dilutions we were only able to determine total bacterial numbers ranging between 10^5^-10^7^ cfu/kg. Product I, III and IV were sold as feed supplements. The declared purpose of use for product I was that it contained “digestive enzymes and useful bacteria to improve feed digestibility in shrimp and to improve the efficiency of feed use”; product III stated that it “provided digestive enzymes to improve feed digestibility in shrimp. Prevention and treatment for white feces/stool disease. Inhibit growth of E. coli, Aeromonas…. enhance immune system. Enhance survival rate, reduce stress”; and product IV stated that it “provided digestive enzymes to improve feed digestibility in shrimp”. Products used as feed supplements stated that 3–10 g/kg feed should be applied between 1 to 4 times per day; however, only two products described how the product should be diluted in water before mixing with the feed and subsequent addition of a binding substance. Products I and IV were also declared to contain phytase, amylase and amylase, protease, respectively. Products II, V, VI and VII used for water treatment stated on the labels that they degraded organic wastes in water and sediment and improved water quality. Two products also stated that they absorbed toxic gases like NH_3_, NO_2_, H_2_S and CH_4_ and one product declared that it would “change the water color from green, dark red to a beautiful water color” as well as increasing oxygen level in water. Product VI also declared to contain a range of different ionic metal compounds, including CuSO_4_. The dose of application varied between 1 kg per 2,000–10,000 m^3^ pond water depending on the concentration of the probiotic strains in the individual product. Products for water treatment was stated to be used in the morning every 5 to 7 days; however, it was not declared whether products should be applied continuously during the entire production cycle.

### Bacterial Species in Probiotic Products

The colonies isolated on blood agar varied in size (0.5 to 6 mm diameter), colour (frost-glass appearance, semi-transparent, white), shape (convex, flat), surface (rough, smooth surface) and margin (irregular margin, fine or regular margin) structures (Table 2). No visible bacterial growth was observed for product II following heat treatment and subculture on blood agar for isolation of *Bacillus* species. As *Lactobacillus* spp. and *Pediococcus acidilactici* were not identified after direct plating on blood agar, MRS agar and APT agar, the analysis of the probiotic samples were subsequently repeated with initial enrichment in 0.9% peptone water overnight at 30°C before subculture on the agars mentioned. However, still only *Bacillus* spp. was isolated. Three to five colonies with identical colony morphology were selected on blood agar for each individual probiotic product yielding a total of 125 isolates selected for subsequent identification by 16S rRNA sequence analysis (Table 2). *Bacillus* spp. was confirmed based on their reactions in the initial phenotypic testing and single cell and spore formation as shown in a phase contrast microscope after methylene blue staining. The molecular16S rRNA sequence analysis of 119 Gram-positive and six Gram-negative bacterial specimens resulted in identification of three genera (*Bacillus*, *Aerococcus* and *Klebsiella*) representing 19 species, i.e. 118 strains of *Bacillus* spp., one *Aerococcus urinaeequi* strain and six strains of *Klebsiella* spp. (Table 2).

Overall, we were unable to isolate all bacterial species declared in the probiotic products. Bacterial species identified, but not declared included several *Bacillus* spp., *Aerococcus urinaeequi* and *Klebsiella* spp. (Table 2). A total of 15 *Bacillus* spp. were identified compared to the 11 species declared. However, only four of the 11 declared species were isolated. Sixteen isolates identified as *Bacillus anthracis* by 16S rRNA sequencing were subjected to *gyr*B gene sequence analysis that confirmed 12 isolates as *B*. *cereus*. The remaining four isolates yielded incomplete sequence data and were identified by MALDI-TOF which confirmed their identity as *B*. *cereus* (99.9% similarity). None of the tested products declared containing *B*. *cereus*, however products I, IV and VII did in fact contain *B*. *cereus*. Products III and V declared *B*. *thuringiensis* on the labels, but we were not able to isolate this species. *Lactobacillus* spp. was declared for products I, IV, VI and VII, but we were unable to isolate any *Lactobacillus* spp. *Pseudomonas denitrificans* was declared for product VI, but could not be recovered (Table 2). As only product IV was found to contain *Aerococcus urinaeequi* and four *Klebsiella* spp., we obtained a new batch from Vietnam of this product for confirmatory analysis. However, as bacterial analysis of this new batch did not reveal any *Aerococcus* spp. or *Klebsiella* spp., we are uncertain if the finding of these two bacterial genera in the first batch were possibly a contamination when the batch of the probiotics was produced.

**Fig 1:**
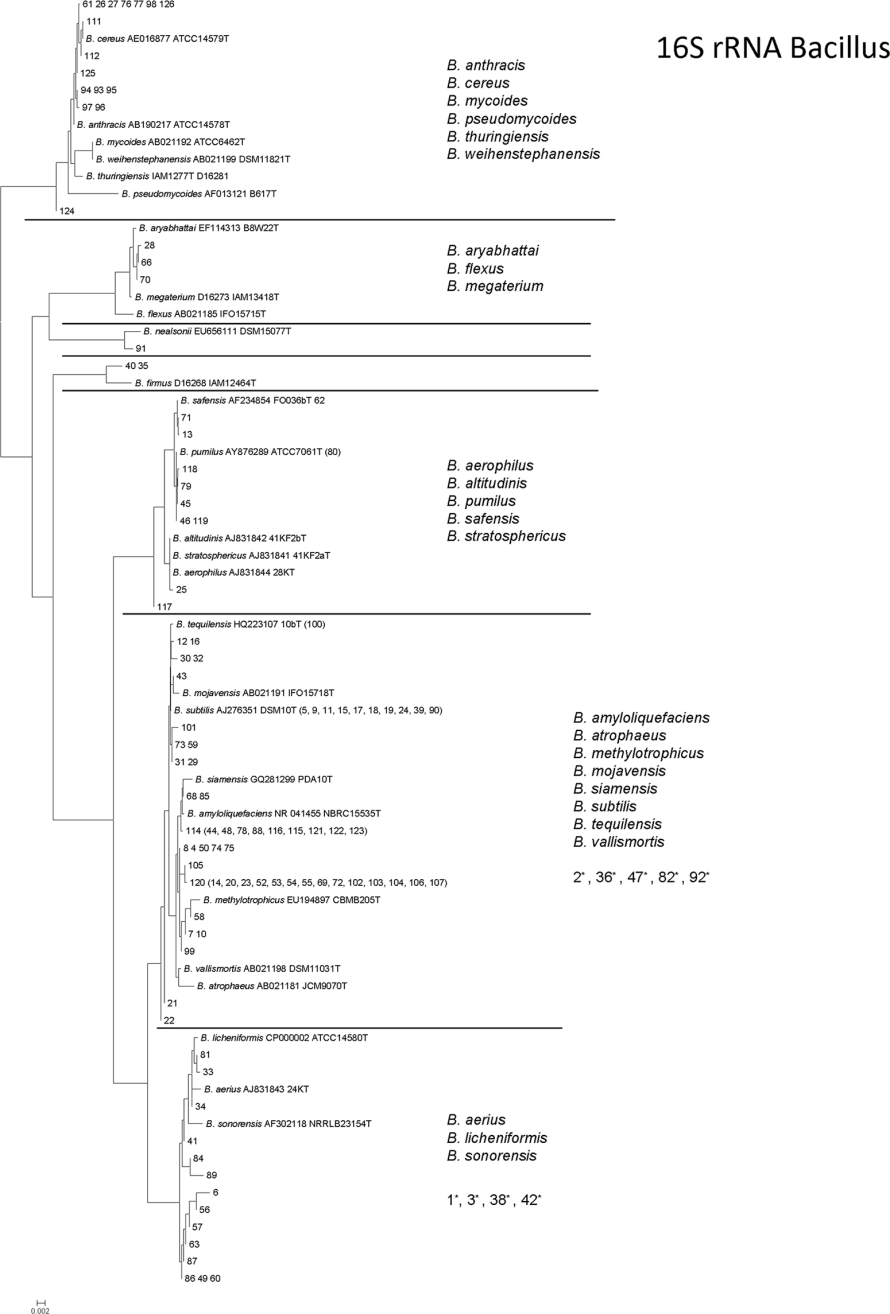
Neighbour joining phylogenetic tree of the 16S rRNA gene sequences representing *Bacillus* spp. isolated from probiotic products. Type strains of *Bacillus* species are labeled with accession number and strain number. Strains with identical sequences are included in parenthesis after the strain selected as reference. Strains with sequences shorter than 1 kb were excluded from phylogenetic analysis and their identity only determined by similarity comparison to type strain. The scale bar represents sequence variation considering the model for nucleotide substitution (Jukes & Cantor) and tree-shape used in the neighbour joining analysis. * Strains are with too short sequences to compare.

Fig 1 shows a phylogenetic analysis based on 16S rRNA gene sequences comparing *Bacillus* spp. isolated from the probiotic products to type strains of the most related *Bacillus* species. Seven groups were observed and five included more than one species of *Bacillus* reflecting their close relationship based on the 16S rRNA gene sequence comparison. The analysis was also used to evaluate the degree of deviation between the declared bacterial species on the product and the species identified in the product.

### Antimicrobial Susceptibility

A total of 65 isolates were selected for antimicrobial susceptibility testing including *Bacillus* spp. (60), *Klebsiella* spp. (4) and *Aerococcus urinaeequi* (1). The antimicrobial resistance patterns are shown in Table 2. In general, the *Bacillus* spp. strains were susceptible to a wide range of the antimicrobials tested, e.g. 9/60 (15%) were fully susceptible to all antimicrobials tested. Twelve (20%) isolates of *Bacillus* spp. were resistant to more than three antimicrobials. Resistance among the *Bacillus* spp. was in particular seen to ampicillin (4), chloramphenicol (18), clindamycin (23), erythromycin (10), and penicillin (11).

The three *Klebsiella* spp. strains were all resistant to ampicillin only whereas the *Aerococcus urinaeequi* strain showed resistance to ampicillin, chloramphenicol, clindamycin, erythromycin, oxacillin, penicillin and sulfamethoxazole/trimethoprim (Table 2).

### Antimicrobial Resistance Genes

The following *Bacillus* spp. strains were selected for whole genome sequencing with the ID number shown in brackets: *B*. *aerius* (42), *B*. *amyloliquefaciens* (8), *B*. *cereus* (26), *B*. *licheniformis* (1), *B*. *nealsonii* (91), *B*. *tequilensis* (11) (Table 2). The analysis of whole genome sequencing data revealed that a macrolide resistance gene *erm*D was found in *B*. *licheniformis* (1) which showed phenotypic resistance to CHL, CLI, ERY, PEN, TET and SXT. *B*. *tequilensis* (11) was found to contain the tetracycline resistance gene *tet*L and showed phenotypic resistance to CHL, ERY, PEN, TET and SXT. The phenicol resistance gene *fex*A and trimethoprim resistant genes *dfr*D, *dfr*G, *dfr*K were identified in *Bacillus nealsonii* (91) which was resistant to CHL, CIP, ERY and SXT. Resistance genes to aminoglycosides, beta-lactams, fluoroquinolones, fosfomycin, fusic acid, macrolide-lincosamide-streptogramin B, rifampin, sulfonamides, tetracyclines, trimethoprim, glycopeptides were not found in *B*. *amyloliquefaciens*, *B*. *aerius*, and *B*. *nealsonii*. No resistance gene of any classes of antimicrobial was identified in *B*. *licheniformis*, *B*. *cereus* and *B*. *tequilensis*. The regions surrounding the resistance genes identified had only similarities to the chromosome of the same species and no similarity to genes previously shown to be involved in horizontal transfer.

## Discussion

### Bacterial Strain Composition

In the present study, 125 bacterial strains were isolated and identified from the selected seven commercial probiotic products commonly used in Vietnamese shrimp culture including 118 strains of *Bacillus* spp., six strains of *Klebsiella* spp. and one *Aerococcus urinaeequi* strain. All seven probiotic products contained bacterial strains that were not declared on the product labels, i.e. a total of 11 *Bacillus* species identified were not declared. Further, seven *Bacillus* species declared could not be isolated (Table 2). In addition, *Lactobacillus* spp. were declared for products I, IV, VI and VII and *Pseudomonas denitrificans* in product VI, however, none of these bacterial species were identified. Standard media and methods were used for the culture of the different declared bacterial species which were subsequently identified by 16S rRNA and gyrase B gene sequence analysis and MALDI-TOF.

We were occasionally able to isolate only less than five colonies of each type seen on the individual culture media despite doing subculture from the lowest dilution of the probiotic product (10^-2^) which indicates a lower concentrations of probiotic strains than declared. Although we find that this do not represent a major bias on the diversity of species identified it may be that some declared species were actually present in the product but that they were not identified, i.e. due to different species showing identical colony morphology. It should be considered to apply culture independent methods, e.g. pyro sequencing, for future determination of bacterial strains in probiotic products.

The initial finding of *Klebsiella* spp. and *Aerococcus urinaeequi* in product IV indicates contamination and inadequate quality control during manufacturing. However, these strains were not isolated when a second batch of product IV was obtained from Vietnam and analysed. It should be noted that we did not identify any *Vibrio* spp., e.g. *V*. *parahemolyticus*, which are associated with the Early Mortality Syndrome (EMS) that currently is causing major mortalities in cultured shrimp all over Asia [5]. As the production of various beneficial compounds and other positive properties of probiotic bacteria are quite strain specific and such properties can vary significantly among strains of the same bacterial species [31], the product labels should correctly state the bacterial species included. However, further details on the actual strains, e.g. a unique identification number, should also be provided allowing users and others to obtain specific information about the specific strains used. None of the products analysed provided information allowing the identification of the specific probiotic strains used.

According to the Vietnamese Ministry of Agriculture and Rural Development (MARD), probiotic products used in aquaculture must be registered before being put on the market, e.g. sold to shrimp farmers. The Directorate of Fisheries under MARD is responsible for the formal approval of probiotic products for use in aquaculture. Such approval is based on performance as documented through on-farm trials, bacterial species composition and concentration analyses performed by laboratories approved by MARD, evaluation of information provided on product labels and on-site inspection of production and storage facilities at the manufacturer [32]. All seven probiotic products analysed in this study were on the list of approved products. It should be noted that the total number of registered products for use in Vietnamese aquaculture in 2012 was 2913, including 813 so-called veterinary drugs and 2100 chemicals, e.g. probiotic products, for water quality improvement and as nutritional supplements [33]. Clearly, the approval of such a high number of registered products according to MARD requirements would demand vast amount of resources and be highly costly. We do not know if the inadequate information provided about the bacterial species included and declared on the products tested in our study may be due to inadequate testing before the products were approved or if bacterial species composition may have been changed after product approval. Also, it seems that a company that wants to market a new probiotic product with an identical bacterial species composition and concentration to an already approved product does not need to document a positive effect in on-farm trials before being put on the market. Our observations of product labels revealed that only two products provided information about how the product should be handled when mixing it with the feed, e.g. need to add a binding substance. Information provided on some labels were unclear and seemed to exaggerate the effectiveness of the product, e.g. product IV was declared to increase oxygen level. Aguirre-Guzman et al. [34] proposed that probiotics marketed for aquaculture use should specify documented effect within bacterial antagonism, competitive exclusion of bacteria, immune stimulation, adhesion properties, improved digestion of feed, and improved pond water quality.

Most of the microorganisms in probiotics used in aquaculture are *Bacillus* spp., *Lactobacillus* spp. and yeast, although other bacterial species like *Nitrosomonas* spp. may also be included [35]. We identified a total of 15 *Bacillus* spp. in the seven products tested and *Bacillus* spp., including *B*. *clausii*, *B*. *licheniformis*, *B*. *cereus*, *B*. *pumilus* and *B*. *thuringiensis*, are often included in probiotic products [36] as they are reported to produce antimicrobial compounds inhibitory to pathogens and stimulate the immune system [7]. Equally important is that *Bacillus* spp. can be kept in the spore form and therefore stored at ambient temperatures for long periods [36]. The problems with misidentification and labelling of *Bacillus* spp. described in the current study are supported by similar observation by Hoa et al. [37] who found that *Bacillus* species used for oral bacteriotherapy and prophylaxis of gastroenteritis were mislabelled as *B*. *subtilis*. Huys et al. [38] reported that more than 28% of the commercial cultures intended for human and/or animal probiotic use were misidentified at the genus or species level. In this study, *B*. *anthracis* was identified in products I, IV and VII based on 16S rRNA sequence analysis. However, the 16S rRNA sequence for *B*. *thuringiensis*, *B*. *cereus*, *B*. *anthracis*, *B*. *mycoides*, *B*. *weihenstephanensis* and *B*. *pseudomycoides* are very similar and subsequent sequence analysis of *gyr*B of twelve *B*. *anthracis* strains and MALDI-TOF characterization of another four presumptive *B*. *anthracis* strains showed that they were in fact *B*. *cereus*.

In order to evaluate the extent of deviation between declared *Bacillus* spp. on the product label and species identified, the actual species of *Bacillus* identified were also compared by phylogenetic analysis. Best correspondences were found in product II and III with all isolates identified to the phylogenetic group. In product VI, isolates were allocated to one more group than the groups that included *B*. *subtilis* and *B*. *licheniformis* labelled on the product. Worse correspondence was found in products I, IV and VII that allowed the identification of *Bacillus* spp. in three out of the seven phylogenetic groups besides of the group with the labelled *B*. *subtilis*. In product V, isolates were allocated to one of the groups with the labelled species (*B*. *licheniformis*) but not to the group with another labelled species (*B*. *thuringiensis*) and three other groups included isolates without labelled species.

### Pheno- and Genotypic Characterization of Antimicrobial Resistance in Probiotic Bacteria

It has been suggested that the use of live bacterial culture as dietary supplements for animal and humans could be a neglected, but important source of antimicrobial resistance genes and possible also pathogens [39]. Several commonly used *Bacillus* spp. has been shown resistant to several antimicrobials such as chloramphenicol, tetracycline, erythromycin, lincomycin, penicillin and streptomycin [37]. *B*. *subtilis*, later reclassified as *B*. *clausii* [40], used as probiotic strain for oral bacteriotherapy was found resistant to chloramphenicol, tetracycline, rifampicin and streptomycin [41]. Our study report for the first time on antimicrobial resistance of *Bacillus* strains in probiotic products used in aquaculture. As expected, the different *Bacillus* species showed different resistance patterns with resistance commonly seen to ampicillin, chloramphenicol, clindamycin, erythromycin and penicillin (Table 2). Despite the wide range of (multi)-resistant phenotypes, the sequence analysis of the whole genome of six selected multiple antimicrobial resistant *Bacillus* spp. only revealed resistance genes in *B*. *licheniformis* (*erm*D), *B*. *tequilensis* (*tet*L) and *B*. *nealsonii* (*fex*A, *dfr*D, *dfr*G and *dfr*K) (Table 3). It should be noted that resistance genes were only found for some of the phenotypic resistances shown in the MIC testing (Tables 2 and 4). More importantly the sequence analysis did not reveal any genetic structures, e.g. integrons and transposons, associated with horizontal gene transfer. It is thus, not known whether the presence of some of these genes in some *Bacillus* strains should be considered intrinsic. It should be noted that antibiotic producing bacteria are a natural source of mechanism of antimicrobial resistance. In particular *Bacillus* spp. are known producers of many different types of antimicrobials, e.g. nicin and subtilin, that is an ability considered a good attribute of these bacteria as probiotics [42]. As a consequence, *Bacillus* spp. probiotic bacterial strains are likely to possess intrinsic mechanism of antimicrobial resistance which may explain the mismatch between the phenotypic resistance and low number of resistance genes found in this study. Furthermore resistance to a specific antimicrobial may require a complex phenotype, not depending on the expression of a single gene.

**Table 4:** Antimicrobial resistance genes determined by whole genome sequencing and subsequent analysis by ResFinder sever 1.4.

Name of the bacteria (ID no.)	Resistance gene	% identity	HSP/Query length	Contig	Position in contig	Predicted phenotype	Accession number in GenBank	Phenotypic resistance
*B*. *licheniformis* (1)	*erm*D	96.30	864/864	NODE_5_length_238348_cov_33.901611	19885..20748	Macrolide resistance	M29832	CHL, CLI, ERY, PEN, TET, SXT
*B*. *tequilensis* (11)	*Tet*L	98.84	1377/1377	NODE_25_length_35112_cov_34.579689	5959..7335	Tetracycline resistance	X08034	CHL, ERY, PEN, TET, SXT
*B*. *nealsonii* (91)	*fex*A	99.79	1428 /1428	NODE_97_length_6543_cov_118.952469	4377..5804	Phenicol resistance	AJ549214	CHL, CIP, ERY, SXT
	*dfr*D	83.14	427/501	NODE_214_length_582_cov_225.872849	178..604	Trimethoprim resistance	Z50141	
	*dfr*G	84.47	483/498	NODE_214_length_582_cov_225.872849	123..605	Trimethoprim resistance	AB205645	
	*dfr*K	84.77	394/492	NODE_214_length_582_cov_225.872849	212..605	Trimethoprim resistance	FN677369	
*B*. *amyloliquefaciens* (8)	-	-	-	-	-	-	-	CHL, CLI, ERY, TET
*B*. *cereus* (26)	-	-	-	-	-	-	-	AMP, CHL, PEN, SXT
*B*. *aerius* (42)	-	-	-	-	-	-	-	CIP, CHL

The genes found in the *Bacillus* spp. are encoding resistance to common drug classes (macrolide, tetracycline, phenicol and trimethoprim) and genes associated with resistance to cephalosporins and quinolones were not detected. Compared with the wide range of resistance genes and mobile elements harboured by the normal aquatic bacterial flora [43], the *Bacillus* spp. included in the tested probiotic products used in Vietnamese shrimp culture seem to contribute with very limited types and numbers of resistance genes. It should be noted that we did not study the presence of plasmids in *Bacillus* spp. which previously have been shown important as reservoirs and vehicles for transferable resistance genes [44].

## Conclusion

All seven probiotic products were approved by the Vietnamese authorities but still contained bacterial strains that were not declared on the product labels, i.e. a total of 11 *Bacillus* species identified were not declared. Further, *Bacillus* spp. and other bacterial species declared could not be isolated. Although our culture-based isolation and identification may have missed a few bacterial species present in the tested products this would represent minor bias, but future studies may apply culture independent identification methods like pyro sequencing. Probiotic product label information should instruct on correct use and dosage as well as allowing identification on the individual bacterial strains included rather than just informing about the bacterial species. The approval of probiotic products needs to be strengthened and should include documentation from scientific-based efficacy trials and that antibiotic resistance is not present in probiotic strains. Overall, the *Bacillus* spp. showed limited phenotypic antimicrobial resistance. Whole genome sequencing of selected multiple antimicrobial resistant *Bacillus* spp. showed that they contained a low number of resistance genes to macrolides, tetracycline, phenicol and trimethoprim, but not any genetic structures associated with horizontal gene transfer. With reference to the recently proposed ranking of public health risks associated with antimicrobial resistance genes found in metagenomic studies, the genes found in our study would be ranked as RESCon7 (lowest risks) [45]. In comparison with natural occurring bacterial species in aquaculture environments, our study documents that the probiotic bacterial strains used in Vietnamese shrimp culture contribute with very limited types and numbers of resistance genes.
